# Coarse-Grained Modeling and Molecular Dynamics Simulations of Ca^2+^-Calmodulin

**DOI:** 10.3389/fmolb.2021.661322

**Published:** 2021-08-24

**Authors:** Jules Nde, Pengzhi Zhang, Jacob C. Ezerski, Wei Lu, Kaitlin Knapp, Peter G. Wolynes, Margaret S. Cheung

**Affiliations:** ^1^ Department of Physics, University of Houston, Houston, TX, United States; ^2^ Center for Theoretical Biological Physics, Rice University, Houston, TX, United States

**Keywords:** calmodulin dynamics, calcium-binding protein, conformational changes, intrinsic disorder, AWSEM, community model

## Abstract

Calmodulin (CaM) is a calcium-binding protein that transduces signals to downstream proteins through target binding upon calcium binding in a time-dependent manner. Understanding the target binding process that tunes CaM’s affinity for the calcium ions (Ca^2+^), or vice versa, may provide insight into how Ca^2+^-CaM selects its target binding proteins. However, modeling of Ca^2+^-CaM in molecular simulations is challenging because of the gross structural changes in its central linker regions while the two lobes are relatively rigid due to tight binding of the Ca^2+^ to the calcium-binding loops where the loop forms a pentagonal bipyramidal coordination geometry with Ca^2+^. This feature that underlies the reciprocal relation between Ca^2+^ binding and target binding of CaM, however, has yet to be considered in the structural modeling. Here, we presented a coarse-grained model based on the Associative memory, Water mediated, Structure, and Energy Model (AWSEM) protein force field, to investigate the salient features of CaM. Particularly, we optimized the force field of CaM and that of Ca^2+^ ions by using its coordination chemistry in the calcium-binding loops to match with experimental observations. We presented a “community model” of CaM that is capable of sampling various conformations of CaM, incorporating various calcium-binding states, and carrying the memory of binding with various targets, which sets the foundation of the reciprocal relation of target binding and Ca^2+^ binding in future studies.

## Introduction

**FIGURE 1:**
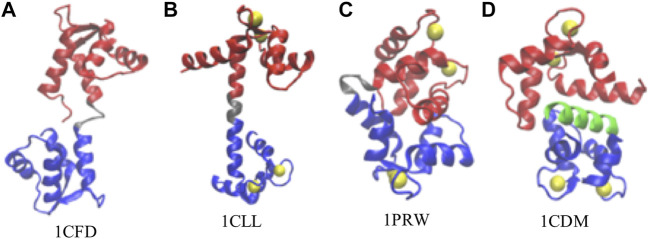
Representative conformations of calmodulin (CaM). **(A)** Ca^2+^-free CaM, or apoCaM (PDB ID: 1CFD); **(B)** Ca^2+^-bound CaM, or holoCaM, in the extended conformation (PDB ID: 1CLL); **(C)** holoCaM in the collapsed conformation without a target peptide (PDB ID: 1PRW); **(D)** holoCaM with a target peptide (PDB ID 1CDM). We visualized the structures using the Visual Molecular Dynamics (VMD) program (Humphrey et al., 1996). Ca^2+^ ions are shown in yellow spheres, the N-terminal and C-terminal domains of CaM are shown in red and blue, respectively, the central linker is shown in grey, and the target peptide is in green.

**FIGURE 2:**
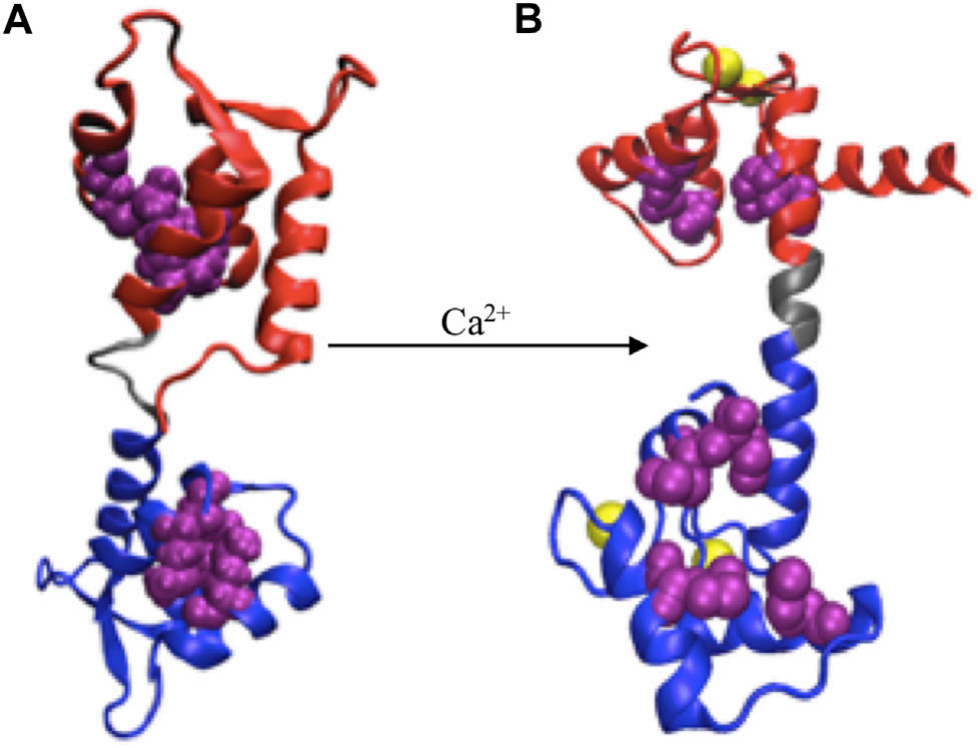
Ca^2+^ induced conformational change. **(A)** apoCaM, **(B)** holoCaM. Ca^2+^ ions are shown in yellow spheres, the N-terminal and C-terminal domains of CaM are shown in red and blue, respectively, and the central linker is shown grey. Upon conformational change, the four methionine residues (shown as beads in purple), initially buried inside the EF-hands, are exposed to the solvent.

Calmodulin (CaM) is a calcium-binding protein that is present in all eukaryotic cells (Berchtold and Villalobo, 2014; Villalobo et al., 2018; Chin and Means, 2000). CaM is composed of two globular domains separated by a central linker. The two domains (N- and C-domains) are constituted of two calcium-binding helix-loop-helix motifs each, namely, EF-hand motifs (Chattopadhyaya et al., 1992; Fernandes and Oliveira-Brett, 2017). Upon sufficient increase in Ca^2+^ concentration, Ca^2+^-free CaM transitions to the Ca^2+^-loaded CaM (Figure 1), exposing the hydrophobic target-binding surfaces of CaM to the solvent (Figure 2) (Barton et al., 2002; Vetter and Leclerc, 2003; Park et al., 2008; Gromiha and Gromiha, 2010; Wu et al., 2012; Fernandes and Oliveira-Brett, 2017). This transition is accompanied by large conformational changes mutually induced by the conformational changes in the CaM-binding target (CaMBT) peptides (Wang et al., 2013; Liu et al., 2017a; Archer et al., 2019; Bekker et al., 2020). The most unique feature of CaM is its reciprocal relation between Ca^2+^ binding and target binding (Meador et al., 1993; Weinstein and Mehler, 1994; Wall et al., 1997; Wriggers et al., 1998; Chin and Means, 2000; Hoeflich and Ikura, 2002; Westerlund and Delemotte, 2018), where the conformational changes in the CaM/CaMBT compound further tune CaM’s affinity for the Ca^2+^ ions (Hoffman et al., 2014; Zhang et al., 2017). The important feature that the net charges of Ca^2+^ ions vary with the conformations of a calcium-binding loop (Zhang et al., 2021), however, has not been accurately captured in molecular dynamics simulations on calcium-binding proteins.

The 148-amino acid CaM is an evolutionarily conserved protein across all vertebrates (Davis and Thorner, 1989; Tripathi et al., 2015a), while it is capable of binding more than 300 variations of CaMBTs (Yamniuk and Vogel, 2004). Such structural variability permits its regulation upon the stimulation of calcium ions processes (Clapham, 2007; Carafoli, 2002; Bootman, 2012) in a wide range of biological activities (Chin and Means, 2000; Hoeflich and Ikura, 2002) including cellular motility, neurogenesis, memory formation, muscle contraction, and neuronal transmission (Bootman, 2012; Marambaud et al., 2009; Berridge, 2006). A large and growing body of literature has investigated the structures and dynamics of CaM upon calcium binding (Wriggers et al., 1998; Mehler et al., 1991; Her et al., 2018; Anthis et al., 2011). The crystal structure of the Ca^2+^-CaM shows a dumbbell-like structure with a central α-helical linker (Figure 1B). However, in solution, the central linker demonstrates higher flexibility (Wriggers et al., 1998; Her et al., 2018; Anthis et al., 2011; Ikura et al., 1992; Chou et al., 2001), which enables the two globular domains of CaM to orient independently from each other (Wriggers et al., 1998; Shepherd and Vogel, 2004; Qin and Squier, 2001). Moreover, this flexibility of the central linker could justify the compact structure of the Ca^2+^-CaM (Figure 1D) upon target binding (Barton et al., 2002; Shepherd and Vogel, 2004). Additionally, Ca^2+^-CaM has been shown to crystallize in the collapsed conformation in the absence of the target (Figure 1C), indeed providing more evidence of the backbone plasticity of the Ca^2+^-CaM (Fallon and Quiocho, 2003) and domain-domain interactions. The structural distribution between the collapsed and extended conformations is roughly 1:9 (Anthis et al., 2011).

A myriad of computational approaches, ranging from all-atom to coarse-grained (Weinstein and Mehler, 1994; Spoel et al., 1996; Wriggers et al., 1998; Vigil et al., 2001; Yang et al., 2001; Barton et al., 2002; Komeiji et al., 2002; Yang et al., 2004; Zuckerman, 2004; Monticelli et al., 2008; Nandigrami and Portman, 2016; Liu et al., 2017b; Gong and Sun, 2017; Robustelli et al., 2018; Delfino et al., 2019; Sun and Kekenes-Huskey, 2021), have been employed to model the structural dynamics of CaM. However, these models have yet to capture the reciprocal relation between Ca^2+^ binding and target binding. The modeling of CaM is often limited for a specific purpose to either bind the target or the calcium ions. As such, CaM is treated as a folded protein (Shepherd and Vogel, 2004; Yang et al., 2004; Zuckerman, 2004) or a folded protein with intrinsically disordered regions (Robustelli et al., 2018), where, in most cases, Ca^2+^ effects are ignored for simplicity (Spoel et al., 1996; Yang et al., 2001; Shepherd and Vogel, 2004; Robustelli et al., 2018). When Ca^2+^ is considered, it is treated as a particle with fixed charges as in an aqueous solution (Monticelli et al., 2008; Gong and Sun, 2017). When Ca^2+^ ions are implicitly included, their charges are often distributed to all the negatively charged residues present in the EF-hand motifs (Nandigrami and Portman, 2016) without any consideration of the coordination geometry of the Ca^2+^ ion, which is influenced by the conformation of a calcium-binding loop (Zhang et al., 2021). Our group has recently proposed a coarse-grained model to study the dynamics of CaM that accounts for both considerations (Wang et al., 2013; Zhang et al., 2017), but it is yet to reproduce the reciprocal relationship between calcium binding and target binding as both require intensive data-driven efforts.

One of the main challenges for developing an appropriate force field involving Ca^2+^ is that CaM is a structurally flexible protein about its central linker while the binding of Ca^2+^ to the two domains of CaM increases the rigidity of the local domains. A compromise between rigidity and softness has to be considered in order for CaM to accurately select, bind, and activate its binding targets. We, therefore, turned to the coarse-grained approach that adopts the Associative memory, Water mediated, Structure, and Energy Model (AWSEM) force field (Wang et al., 2011; Zhang et al., 2017) to fulfill these requirements. The next challenge is to properly include the Ca^2+^ ions in the force field of a coarse-grained model since this would allow us to understand the reciprocal relationship between calcium binding to CaM and binding of a target (Wang et al., 2013; Tripathi et al., 2015b). Although in separate studies, there have been development in AWSEM force fields on structurally flexible proteins (Wu et al., 2018) or on di-valent ions (Tsai et al., 2016), both features, however, have yet to be combined for modeling CaM. Particularly, the coordination chemistry of Ca^2+^ ions and the structural flexibility allowing CaM to flex according to its binding targets and surrounding environment is important in modeling the adaptive behavior of CaM (Wang et al., 2013; Liu et al., 2017a; Archer et al., 2019; Bekker et al., 2020). By taking into account the coordination chemistry of the Ca^2+^ ions as well as the flexible nature of the central linker of CaM, our proposed model for Ca^2+^-CaM elucidates the many-body effects of calcium binding on the geometrical shape of the calcium-binding loop and allows large conformational changes for binding various targets.

## Materials and Methods

### Coarse-Grained Modeling Using the AWSEM Force Field

We used the AWSEM force field (Davtyan et al., 2012; Tsai et al., 2016) to build the model of Ca^2+^-CaM. AWSEM is a transferable coarse-grained protein model, which uses three beads ($C_{\alpha }$, $C_{\beta }$, and O atom from the peptide bond) to represent each amino acid residue (except for glycine, which lacks $C_{\beta }$). The positions of all the rest of the other atoms along the backbone are deduced by assuming an ideal geometry of the system. The total energy function used in AWSEM is given in Eq. 1,

$$V=V_{BB}+V_{PMF}+V_{DH}+V_{FM}$$ (1)

which is composed of two main terms: the physics-based terms, $V_{BB}+V_{PMF}+V_{DH}$, and the knowledge-based term, $V_{FM}$.

(a) The backbone term, $V_{BB}$, from Eq. 1, maintains the backbone geometry of protein. Its expression is given in Eq. 2,

$$V_{BB}=V_{con}+V_{chain}+V_{\chi }+V_{rama}+V_{excl}.$$ (2)

$V_{con}$ and $V_{chain}$ represent the connectivity and chain terms, respectively. The former links neighboring residues, while the latter maintains ideal bond angles around the $C_{\alpha }$ atoms of each residue. The chirality potential, $V_{\chi }$, which is applied to all residues except for glycine, maintains the local residue chirality. $V_{rama}$ represents the Ramachandran potential, which biases the protein chain conformation toward the allowed Ramachandran regions. The excluded volume potential, $V_{excl}$, is used to avoid chain collapse and unphysical entanglements.

(b) The many-body potential of mean force $V_{PMF}$, from Eq. 1, takes into account the nature of the interacting residues, and its expression is given in Eq. 3:

$$V_{\text{PMF}}=V_{\text{contact}}+V_{\text{burial}}+V_{\text{HB}}$$ (3)

The contact term $V_{\text{contact}}$ represents the pairwise additive and the many-body terms that consider interactions between residues far apart in sequence. The burial term $V_{\text{burial}}$ accounts for a particular residue’s type propensity. The hydrogen bond term $V_{\text{HB}}$ is composed of three other terms given in Eq. 4:

$$V_{HB}=V_{\beta }+V_{P-AP}+V_{helical},$$ (4)

where $V_{\beta }$ and $V_{P-AP}$ are for the hydrogen bonding interactions in β-sheet conformations. The former stabilizes already formed β hydrogen bonds, while the latter allows a protein to undergo parallel and anti-parallel β-sheet conformations before the stabilization of the hydrogen bond. The helical term $V_{helical}$ ensures the formation of α-helices.

(c) The electrostatic interactions in solution with implicit solvent are described by the Debye–Hückel term (Tsai et al., 2016) $V_{DH}$ from Eq. 1. The expression is given in Eq. 5:

$$V_{DH}=K_{Elec}\underset{i<j}{\sum }\frac{q_{i}q_{j}}{\varepsilon_{r}r_{ij}}\text{exp}\left(-\frac{r_{ij}}{l_{D}}\right),$$ (5)

where $q_{i}$ and $q_{j}$ represent the charges of beads i and j; $r_{ij}$ is the distance between the two beads; $K_{Elec}={\left(4\pi \varepsilon_{0}\right)}^{-1}=332.24\;kcal\;mol^{-1}e^{-2}Å$; $\varepsilon_{r}$ represents the dielectric constant of the media; and $l_{D}$ is the Debye–Hückel screening length, which is given by lD=εrε0kBT2e2I. $k_{B}$ is the Boltzmann constant, T is the temperature, e represents the elementary electric charge, and I is the ionic strength of the implicit solvent.

(d) $V_{FM}$ from Eq. 1, is a bioinformatic fragment memory potential that structurally biases short fragments of the protein chain, typically composed of 3–9 residues at a time, towards conformations that are based on “memory” structures. As listed in Table 1, the memory structures selected from the Protein Data Bank (PDB) were used to speed up the conformational search of the native states. Its expression is given in Eq. 6:

$$V_{FM}=-\lambda_{FM}\overset{60}{\underset{m=1}{\sum }}.\overset{3}{\underset{n=1}{\sum }}W_{n}^{m}\underset{i,j∋3\leq |i-j|\leq 9}{\sum }\gamma_{ij}exp\left[-\frac{{\left(r_{ij}-r_{ij}^{m}\right)}^{2}}{2\sigma_{ij}^{2}}\right],$$ (6)

where the outer sum is over the aligned memory (from m=1 to 60), the sum in the middle with a variable n is over the three segments of CaM (n=1 is N-domain, n=2 is central linker, and n=3 is C-domain), and the inner sum is over all possible pairs of $C_{\alpha }$ and $C_{\beta }$ atoms within the memory fragments that are separated by two or more residues; |i−j|≤9 gives the maximum sequence separation of interacting residues, which is either the length of the memory or the maximum cutoff, whichever is shorter. $r_{ij}$ and $r_{ij}^{m}$ represent the instantaneous distance and the corresponding distance in the memory fragment between the atoms i and j; $\gamma_{ij}$ is the residue type dependence interaction strength; $\sigma_{ij}$ represents the sequence separation dependent width. Its expression is given by $\sigma_{ij}=|i-j{|}^{0.15}$.

**TABLE 1:** The PDB IDs of the 60 non-redundant CaM memory structures for the AWSEM model and the Rg of the CaM. In the case of CaM complexes, only the coordinates of CaM were used for memory. *m* is the index for the memories.

*m*	PDB ID	Rg (Å)	*m*	PDB ID	Rg (Å)	*M*	PDB ID	Rg (Å)
1	1PRW	14.60	21	3GP2	15.96	41	2BBM	17.03
2	3EWT	15.17	22	3DVM	16.00	42	2BCX	17.25
3	2LGF	15.23	23	1CKK	16.05	43	2KNE	18.67
4	3DVJ	15.31	24	2FOT	16.05	44	4DCK	18.87
5	3BYA	15.38	25	2JZI	16.12	45	1SK6	19.30
6	2LHI	15.38	26	1CM1	16.20	46	1XFU	19.31
7	2HQW	15.41	27	3DVK	16.22	47	2L1W	19.46
8	2L7L	15.43	28	2VAY	16.26	48	1K9O	19.46
9	3EWV	15.52	29	2O60	16.28	49	1PKO	19.49
10	1IWQ	15.57	30	2WEL	16.35	50	1S26	19.50
11	1L7Q	15.59	31	1QTX	16.43	51	1LVC	19.70
12	3DVE	15.61	32	2O5G	16.47	52	1CFF	19.78
13	1CDM	15.66	33	1QS7	16.50	53	4G27	20.79
14	3SUI	15.68	34	3GOF	16.50	54	2L53	21.00
15	3BXL	15.80	35	3BXK	16.51	55	2KDU	25.65
16	1IQ5	15.90	36	1MXE	16.55	56	4EHQ	22.07
17	1ZUZ	15.93	37	2Y4V	16.62	57	4DJC	22.31
18	3HR4	15.94	38	2BE6	16.65	58	2YGG	23.66
19	2KOF	15.95	39	2F3Y	16.68	59	1G4Y	25.32
20	2LL6	15.95	40	1SY9	16.83	60	1CLL	21.80

We used 60 non-redundant structures of CaM taken from our prior study (Tripathi et al., 2015c) (Table 1), either free or in complex with the target peptides, to build the memory fragments for the coarse-grained model of CaM. We used the 60 sequence homologs of Ca^2+^-CaM since we are interested in studying the dynamics and the folding properties of CaM. We divided each memory into three segments: N-domain of CaM, from residue 5 to residue 76; the flexible central linker, from residue 77 through residue 81; and C-domain of CaM, from residue 82 to residue 147 (as illustrated in Figure 1). The weight of each segment is controlled by a memory weight parameter $W_{n}^{m}$ and a global parameter $\lambda_{FM}$ (Eq. 6). Because the central linker has its own memories of large variations in the structures, essentially it was modeled with more structural flexibility than the two globular domains of CaM.

We summarized CaM models using various values of $\lambda_{FM}$ and $W_{n}^{m}$ parameters in Table 2 for our molecular simulations. All other parameters for AWSEM were set by default as defined in the original ASWEM study (Davtyan et al., 2012; Tsai et al., 2016). The parameters of the default setting are also provided in the Supplemental Information (SI 1).

**TABLE 2:** Summary of Ca^2+^-CaM models with different values of the global and local memory weight and the average Rg ($\bar{\mathrm{Rg}}$) and the ratio between the collapsed and extended states (γ_c:e_) from the umbrella sampling simulations. The models that best reproduce experimental results from our simulations are marked with *. The experimental measured Rg = 20.0 ∼ 22.5 Å and γ_c:e_ ∼ 0.11.

Models	$\lambda_{FM}$	$W_{n}^{m}$				$\bar{\mathrm{Rg}}$ (Å)	γ_c:e_
		$W_{n}^{\text{others}}$	$W_{1}^{1\text{CLL}}$	$W_{2}^{1\text{CLL}}$	$W_{3}^{1\text{CLL}}$		
I.1	1	1	1	1	1	22.05	0.001
I.2	0.1	1	1	1	1	20.51	0.48
I.3	0.01	1	1	1	1	19.86	0.64
II.2	0.1	1	2	2	2	20.34	0.59
II.3	0.1	1	3	3	3	20.66	0.47
II.4	0.1	1	4	4	4	20.43	0.50
II.5	0.1	1	5	5	5	20.70	0.37
II.6	0.1	1	6	6	6	20.52	0.38
II.7	0.1	1	7	7	7	21.11	0.16
*II.8	0.1	1	8	8	8	21.15	0.14
*II.9	0.1	1	9	9	9	21.26	0.10
III.1	0.1	1	5	1	5	19.91	0.73
III.2	0.1	1	5	2	5	19.70	0.82
III.3	0.1	1	5	3	5	19.84	0.77
III.4	0.1	1	5	4	5	20.03	0.58
III.6	0.1	1	5	6	5	20.43	0.48
III.7	0.1	1	5	7	5	21.20	0.16
*III.8	0.1	1	5	8	5	21.35	0.12
III.9	0.1	1	5	9	5	21.59	0.07

### Modeling Ca^2+^ Ions in ASWEM Force Field

**FIGURE 3:**
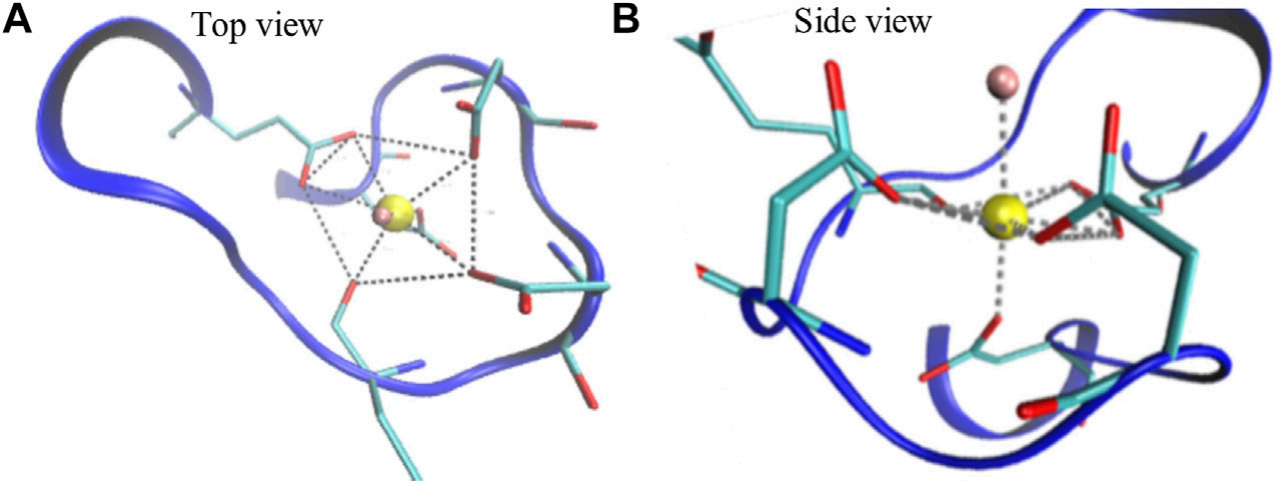
Illustration of a Ca^2+^ binding loop at the fourth EF-hand of CaM (PDB ID: 1CLL). The pentagonal bipyramidal geometry of the Ca^2+^ coordination is represented with the dotted lines in the top view (**A**) and side view (**B**). The red sticks linked with the dotted lines represent oxygen atoms that participate in coordination. The water molecule, shown in pink, completes the spherical coordination through hydrogen bonding.

Calcium-binding proteins in most cases contain the EF-hand calcium-binding motif which is formed by the pentagonal bipyramidal geometry (Figure 3; Supplementary Figure S1 in the SI) (Drake et al., 1997; Yang et al., 2002). The literature on modeling those proteins, in general, does not clearly explain how Ca^2+^-binding effects are considered during the simulations. We developed an implicit Ca^2+^ model that evenly splits the Ca^2+^ charges to a selection of residues. Partial charges on the sidechain beads of the negatively charged residues that coordinate Ca^2+^ collectively in the Ca^2+^ binding loops (Figure 3) were adjusted to reflect the Ca^2+^- binding effect (Table 3). To note, charges on those acidic residues which do not coordinate (Ca^2+^) remain intact (−1 e). This simple approach incorporates the coordination geometry of the Ca^2+^ and allows us to improve the modeling of many-body effects in the local Ca^2+^ binding loops which furthermore controls the open/close state of the helix-loop-helix EF-hand and the global conformation of calmodulin.

**TABLE 3:** The adjusted partial charges of the negatively charged residues participated in calcium coordination in the four calcium-binding loops of CaM. The amino acid sequences of the calcium-binding loops are provided as a reference. The subscript indices stand for the positions of the starting/ending residues in the CaM sequence. Underscored residues are the selected amino acids with negative charges as shown in the third column. We adjusted the partial charges on the sidechain beads of those residues based on the bipyramidal pentagonal coordination geometry of the calcium ion.

Calcium loop	Amino acid sequence	Adjusted partial charges on selected amino acids
Loop 1	_20_DKDGDGTITTKE_31_	-0.5, -0.5, -0.5, -0.5
Loop 2	_56_DADGNGTIDFPE_67_	-0.5, -0.5, -0.5, -0.5
Loop 3	_93_DKDGNGYISAAE_104_	-0.33, -0.33, -0.33
Loop 4	_129_DIDGDGQVNYEE_140_	-0.5, -0.5, -0.5, -0.5

In this study, we compared the abovementioned approach (approach III) with two other calcium models:

I. Splitting the Ca^2+^ charges evenly to the neighboring negatively charged residues (Tsai et al., 2016).

II. Splitting the Ca^2+^ charges evenly to the residues that participate with the calcium coordination and the radius of gyration of the sidechain beads are constrained by a stiff harmonic potential with a force constant of 30 kcal/mol/Å^2^.

III. The same with approach II except that the harmonic constraint is removed.

Comparing to approach I of Ca^2+^ in AWSEM (Tsai et al., 2016), in approach III calcium charges were split according to the coordination chemistry of calcium; comparing to approach II, in approach III more degrees of freedom to the Ca^2+^ coordination residues allow sampling of more different conformations of CaM.

### Simulation Details

We performed the coarse-grained simulations with the open-source MD package LAMMPS, in which AWSEM code was implemented (Davtyan et al., 2012). We used the periodic boundary conditions on the cubic box of 400 Å on each side so that even unfolded CaM can fit in the box. Initial velocities were chosen randomly from a Boltzmann distribution with the average squared velocity equal to 3 ${\text{k}}_{B}$T/m, where ${\text{k}}_{B}$ is the Boltzmann constant, m is the mass, and the temperature is T. We also used the cutoff distance of 3.5 Å and κ (inverse of the Debye length) = 0.0127 Å^−1^ in the electrostatic term.

Simulated annealing: firstly, we denatured the protein at 450 K using the initial structure built from the crystal structure (PDB ID: 1CLL) (Chattopadhyaya et al., 1992); then, we performed simulated annealing on Ca^2+^-CaM by cooling down the system to a low temperature (280 K) for 2,000,000 time steps for each value of $\lambda_{FM}$ (models I.1–3 in Table 2).

Production simulation: we employed the umbrella sampling (US) method (Roux, 1995) to evaluate the thermodynamic properties of Ca^2+^-CaM using different memory parameters. We conducted the production simulations in canonical ensemble (NVT) at the constant temperature of 300 K. Radius of gyration (Rg) of Ca^2+^-CaM was used as the reaction coordinate, which was restrained by a harmonic potential $E_{Restr}^{i}=\frac{1}{2}\text{k}{\left({\text{Rg}}^{\text{i}}-{\text{Rg}}_{0}^{\text{i}}\right)}^{2}$ with a force constant k = 50 kcal/Å^2^. The equilibrium positions of the harmonic potential ${\text{Rg}}_{0}$ range from 13.00 Å to 26.00 Å with a bin size of 0.25 Å, making up a total of 53 windows (i = 0, 1, 2, …, 52). The setup of the force constant and the equilibrium positions of the windows is justified by the fluctuations of Rg around the corresponding equilibrium positions as well as the overlap in the Rg distribution between neighboring windows, which are provided in Supplementary Figures S4–S5, respectively, in the SI. For each window, 2,000,000 time-steps of constrained molecular simulations were conducted using different initial conditions. To generate the initial structures at each window for the US simulations, molecular dynamics simulations were carried out for the Ca^2+^-CaM from the crystal structure of CaM (PDB ID: 1CLL, in the coarse-grained model) at a temperature T = 300 K. Structures with Rg closest to ${\text{Rg}}_{0}^{\text{i}}$ were selected as the initial configurations. A set of 5 US simulations were carried out at each window with the same initial configuration and different random initial velocities. The integration time step is 2 fs. It is important to note that with the smoothed CG potential, the realistic time represented by a time step is longer than 2 fs. Coordinates and Rg were recorded every 1,000 time steps for analyses, making up 10, 000 frames of data for analysis at each window. We analyzed the data using a multi-state Bennett acceptance ratio (MBAR) estimator with the *pymbar* program (Shirts and Chodera, 2008).

### Data Analysis

We described the conformational changes of the Ca^2+^-CaM during the simulations by calculating the radius of gyration (Rg) and the pairwise comparison (${\text{Q}}_{w}$) from the crystal structure (PDB ID: 1CLL).

#### Radius of Gyration (Rg)

Rg describes the compactness of the protein, and its definition is given in Eq. 7:

$$\text{R}\text{g}=\sqrt{\frac{\sum_{i=1}^{N}{\text{m}}_{i}\Delta r_{i}^{2}}{\sum_{i=1}^{N}{\text{m}}_{i}}}.$$ (7)

In the case of PDB structures, ${\text{m}}_{i}$ is the mass of the $i^{th}$ atom and $\Delta {\text{r}}_{i}$ is the distance between the $i^{th}$ atom and the center of mass of the system. In the case of coarse-grained structures, only the C_α_ beads are used, ${\text{m}}_{i}$ is the mass of the C_α_ bead of the $i^{th}$ residue, and $\Delta {\text{r}}_{i}$ is the distance between the C_α_ bead of the $i^{th}$ residue and the center of mass of the system.

#### Pairwise Comparison

$Q_{w}$ measures the degree of similarity of conformations from the trajectories with respect to the native structure through the pairwise comparison. In other words, it compares the pairwise distances of $C_{\alpha }$ atoms (beads) among the residues in each instantaneous structure to counterpart in the native structure. This value is normalized to 1, with a higher value corresponding to the greater similarity to the native structure. In this case, $Q_{w}$ is the extended crystal structure of Ca^2+^-CaM (PDB ID: 1CLL), $Q_{w}$ = 1. The expression of the order parameter $Q_{w}$ is given in Eq. 8:

$$Q_{w}=\frac{2}{\left(N-2\right)\left(N-3\right)}\underset{|j-i|\geq 2}{\sum }exp\left[-\frac{{\left(r_{ij}-r_{ij}^{m}\right)}^{2}}{2\sigma_{ij}^{2}}\right],$$ (8)

where N is the total number of residues, $r_{ij}$ represents the instantaneous distance between $C_{\alpha }$ atoms (beads) of residues *i* and *j*, $r_{ij}^{m}$ is the same distance in the reference structure (PDB ID: 1CLL), and $\sigma_{ij}$ is obtained from the following relation $\sigma_{ij}={\left(1+|i-j|\right)}^{0.15}$.

#### Definition of Collapsed and Extended Conformations of CaM

**FIGURE 4:**
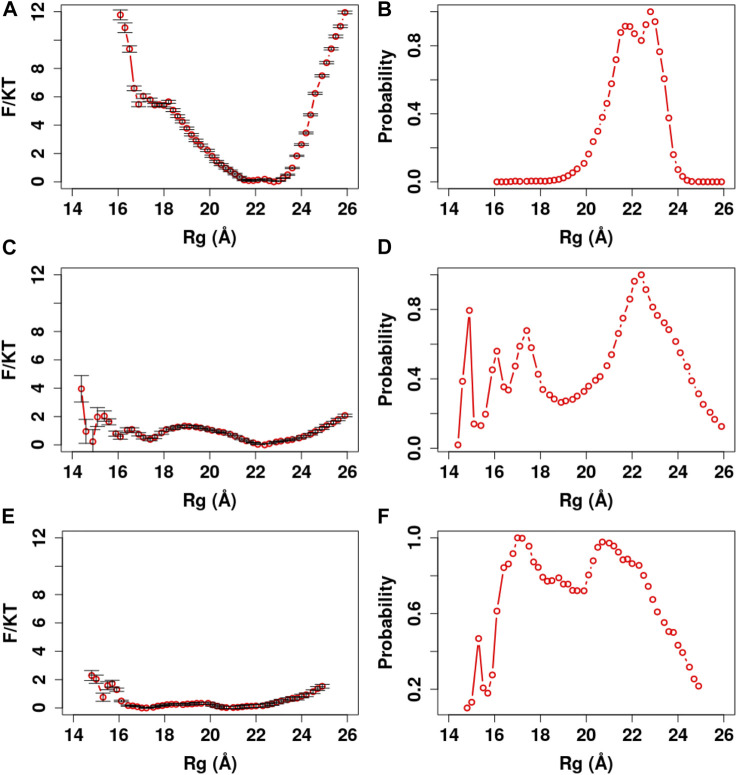
PMF and Rg distribution of the Ca^2+^-CaM models using the global memory weight of different order of magnitudes. The memory weight parameters of the Ca^2+^-CaM models are listed in Table 2 (model I; $\lambda_{FM}$ = 1, 0.1, 0.01, and $W_{n}^{\text{m}}$ = 1). **(A, C, E)** The reweighted free energy profile as a function of Rg from US simulations with $\lambda_{FM}$ = 1, 0.1, 0.01, respectively. (B, D, F) The probability distribution as a function of Rg. The standard errors in the PMF are provided.

**FIGURE 5:**
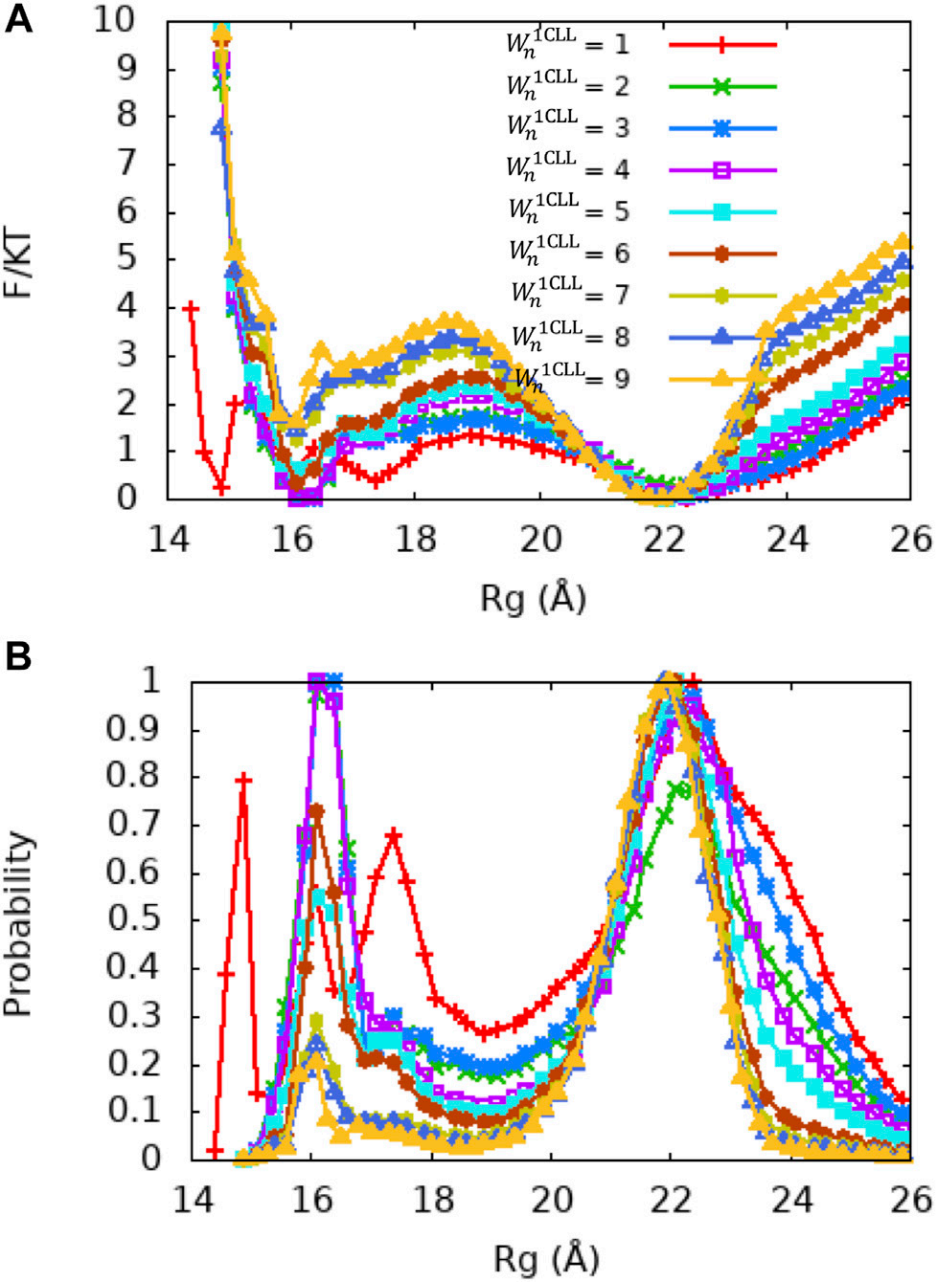
PMF and Rg distribution of the Ca^2+^-CaM models for different values of the local memory weight $W_{n}^{1\text{CLL}}$ (n = 1, 2, 3). The memory weight parameters of the Ca^2+^-CaM models are listed in Table 2 (model II; ${\text{λ}}_{FM}=0.1$, $W_{n}^{\text{others}}$ = 1, and $W_{n}^{1\text{CLL}}=1-9$). **(A)** The reweighted free energy profile as a function of Rg from US simulations **(B)** and the probability distribution as a function of Rg. The standard errors are too small to show and are provided in Figures S6 in the SI.

**FIGURE 6:**
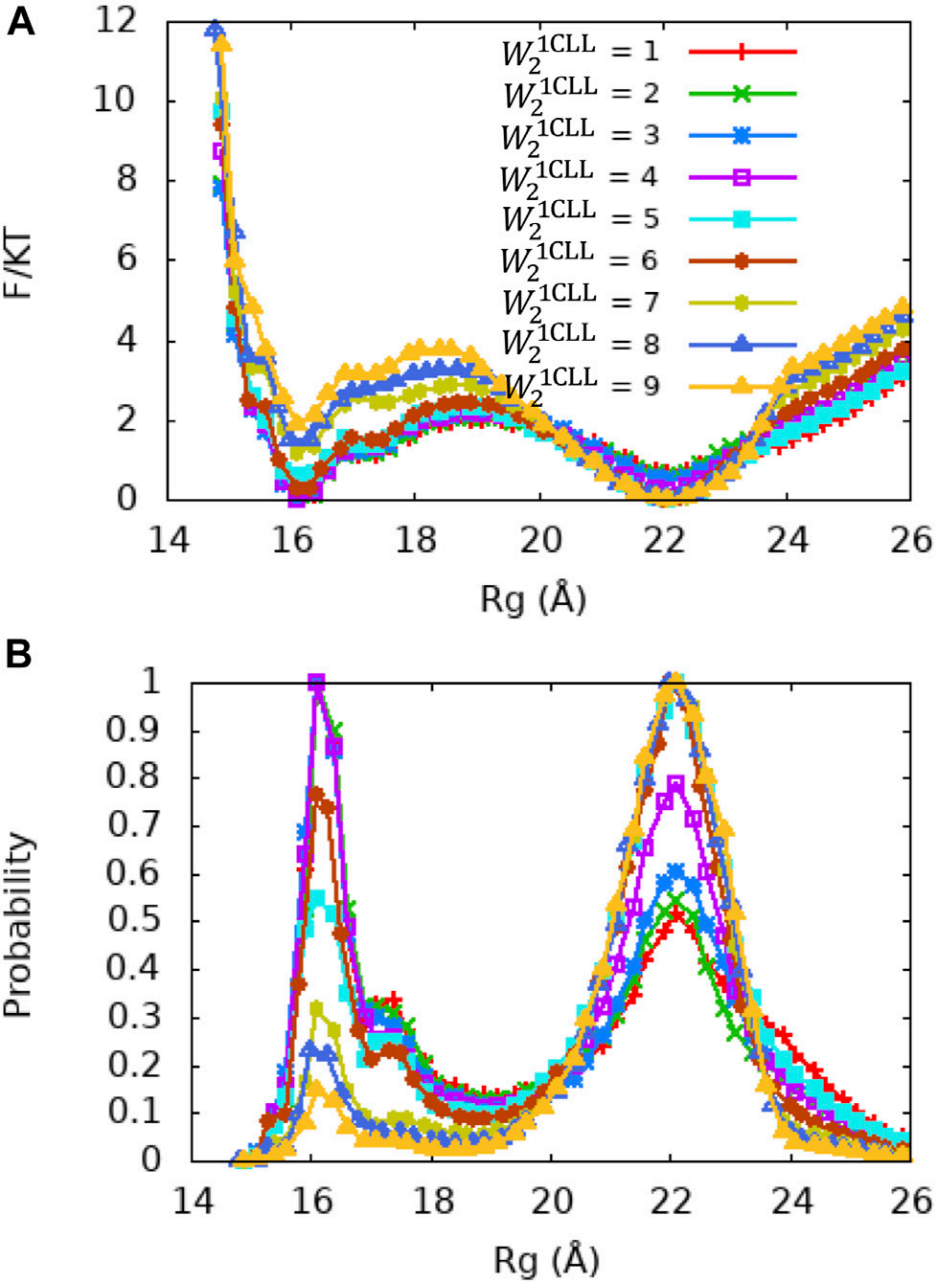
PMF and Rg distribution of the Ca^2+^-CaM models for different values of the local memory weight $W_{n}^{1\text{CLL}}$ (n = 2) for the central linker of CaM. The memory weight parameters of the Ca^2+^-CaM models are listed in Table 2 model III; ${\text{λ}}_{FM}=0.1$, $W_{n}^{\text{others}}$ = 1, $W_{1,3}^{1\text{CLL}}=5$, $W_{2}^{1\text{CLL}}=1-9$. **(A)** The reweighted free energy profile as a function of Rg from US simulations **(B)** and the probability distribution as a function of Rg. The standard errors in the PMF are provided in Figure S7 in the SI.

**FIGURE 7:**
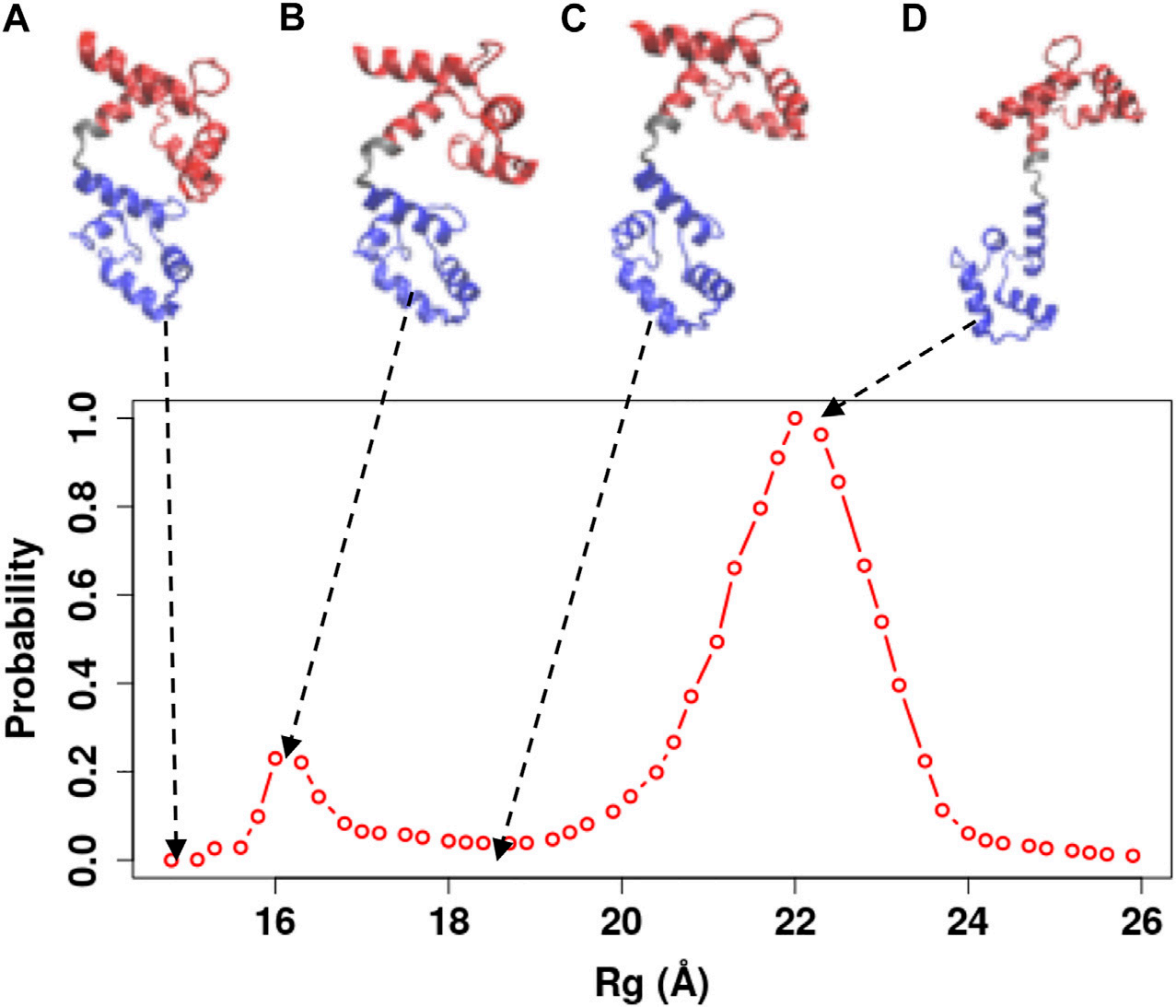
Representative snapshots of the Ca^2+^-CaM model obtained from our simulations. The model that best reproduces experimental measurements was used (${\text{λ}}_{FM}=0.1$, $W_{n}^{\text{others}}$ = 1, $W_{1,3}^{1\text{CLL}}=5$, and $W_{2}^{1\text{CLL}}=8$). **(A)** The completely collapsed conformation of the Ca^2+^-CaM; **(B)** the collapsed conformation; **(C)** the intermediate conformation; and **(D)** the extended conformation. These structures are represented along the probability distribution of Rg.

**FIGURE 8:**
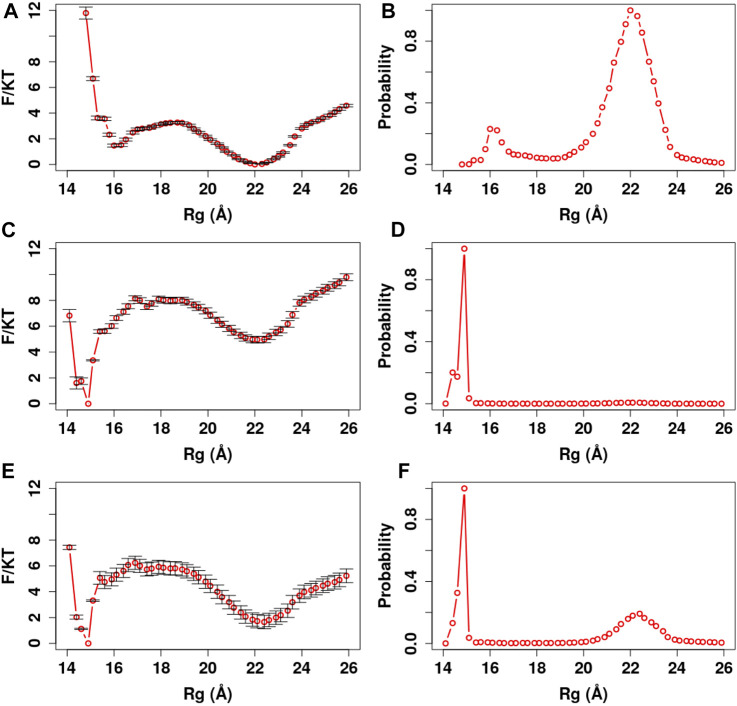
PMF and Rg distribution of the Ca^2+^-CaM for different Ca^2+^ models. The optimized memory parameters for the Ca^2+^-CaM model were used (${\text{λ}}_{FM}=0.1$, $W_{n}^{\text{others}}$ = 1, $W_{1,3}^{1\text{CLL}}=5$, and $W_{2}^{1\text{CLL}}=8$). The three charge models are described in the *Method* section. **(A)** The reweighted free energy profile as a function of Rg from the US simulations **(B)** and the probability distribution as a function of Rg. The standard errors in the PMF are provided.

In our analyses, we used the parameter Rg to define a collapsed or extended conformation. The Rg cutoff was set according to the potential of mean force (PMF) and distribution of Rg in our simulations (Figures 4–8). A collapsed conformation is defined if Rg < 18.5Å; otherwise, an extended conformation is defined.

## Results and Discussion

The goal is to create a computational model for CaM accommodating its several key functions including divalent ion binding, conformational dynamics, and target recognition. In order to achieve this, we adopted a pool of 60 CaM or CaM complex structures from our previous study (Tripathi et al., 2015c) representing full-length CaM without mutations and its complex with 24 unique target proteins/peptides. As stated in the *Materials and Methods Simulation Details*, our approach is to tune the two scaling parameters $\lambda_{FM}$ and $W_{n}^{m}$ to adjust the global and each segment of the multiple memories, respectively, relative to other terms in the total energy function. After optimizing the memory parameters, three models of calcium are compared to describe the divalent ion binding.

Each of the 60 memories has a specific conformation of calmodulin which could be helpful in target recognition and target selection; therefore, clustering the 60 memories may leave out important conformations that accommodate recognition of a specific target protein. Potentially, there could be as many as 181 fragment memory parameters to be determined in the most complicated scenario. However, in the general practice of AWSEM modeling, a search in the available determined structures in the database according to the match in the desired amino acid sequence is conducted to generate a library of memory structures. Therefore, to avoid excessive tuning of the parameters, the individual memory weight W_n_^m^ is kept as a constant default value. Because the unbound extended form of the Ca^2+^/CaM (PDB code: 1CLL) was found to be most dominant experimentally, we adjusted its local memory weight parameter correspondingly, while keeping others the default $W_{n}^{others}=1$. The actual parameters we tuned eventually reduce to $\lambda_{FM}$ and $W_{n}^{1CLL}(n=1,\;2,\;3$ to represent N-domain, central linker, and C-domain of the protein).

Parameterization of our CG model was guided by the existing following experimental measures: Rg of Ca^2+^-CaM spans from 20.0 to 22.5 Å measured by small angle X-ray scattering (SAXS) experiments (Seaton et al., 1985; Heidorn and Trewhella, 1988; Kataoka et al., 1991); in addition, the population of CaM’s collapsed conformation is about 10% measured by nuclear magnetic resonance (NMR) experiments (Anthis et al., 2011) (i.e., the ratio between the population of the collapsed conformation and that of the extended population is $\gamma_{c:e}∼0.11$).

### The Global Parameter Weight Controls the Energy Barrier Between Extended and the Collapsed States of Ca^2+^-CaM

Firstly, we determined the magnitude of the fragment memory interaction by investigating its effect on the folding properties of Ca^2+^-CaM. We carried out simulated annealing using the unfolded structure of the Ca^2+^-CaM ($Q_{w}∼0.2$ as shown in Supplementary Figure S2 in the SI) with three orders of magnitudes of the global scaling parameter $\lambda_{FM}$ = 1, 0.1, 0.01. At $\lambda_{FM}=1$, the system transitions abruptly from the unfolded to the folded state (Supplementary Figures S3A, S3B) in the SI; at $\lambda_{FM}=0.1$, smooth transitions of Ca^2+^-CaM from the unfolded to the folded structure were observed with $Q_{w}$ reaching a maximum value of 0.67 (Supplementary Figures S3C, S3D) in the SI; at $\lambda_{FM}=0.01$, the maximum value of $Q_{w}$ was less than 0.55 and the total energy of the system did not stabilize on a long timescale (Supplementary Figures S3E, S3F) in the SI.

In addition, we carried out US simulations to evaluate thermodynamic properties of Ca^2+^-CaM with the global scaling parameter in three orders of magnitudes $\lambda_{FM}$ = 1, 0.1, and 0.01, while keeping the local memory scaling parameters $W_{n}^{m}$ = 1. The PMF and the probability distribution as a function of Rg were computed using the MBAR estimator (Figure 4) and the average Rg ($\bar{\text{Rg}}$) and the ratio between the population of collapsed conformation and that of the extended conformation (γ_c:e_) were evaluated from the PMF. For $\lambda_{FM}=1$, Ca^2+^-CaM mostly samples the extended conformations (with Rg ∼ 21.5 to 22.5 Å) and the free energy barrier between the extended and collapsed conformations is quite large (∼6 k_B_T); therefore, the system lacks the capacity of shifting between multiple conformations. For $\lambda_{FM}=0.1$, there are four minima in the PMF of Ca^2+^-CaM with Rg ranging from 15 to 22 Å (Figure 4), and the free energy barrier between the collapsed states and the extended state is ∼ k_B_T; the $\bar{\text{Rg}}$ of Ca^2+^-CaM is within the experimental range ($\bar{\text{Rg}}\;$= 20.51 Å); however, γ_c:e_ (∼ 0.48) is much higher than the experimental one (∼ 0.11) (Anthis et al., 2011). It is worthwhile to note that the minimum at Rg ∼ 15 Å resembles a completely collapsed structure as found in our previous study (Homouz et al., 2009). For $\lambda_{FM}=0.01$, there are noticeably two minima located at Rg ∼ 17 Å and Rg ∼ 20.5 Å, sampling a somewhat collapsed conformation and a less extended conformation (comparing with $\lambda_{FM}$ = 1, 0.1) respectively with free energy barrier < k_B_T. $\bar{\text{Rg}}$ = 19.86 Å, below the experimentally measured Rg range (Seaton et al., 1985; Heidorn and Trewhella, 1988; Kataoka et al., 1991) and γ_c:e_ (∼ 0.64) is much higher than the experimental value (Anthis et al., 2011).

In summary, the global memory parameter $\lambda_{FM}$ is effective for controlling the sampling of the collapsed and extended conformations of CaM as well as reproducing the averaged Rg. A large value of the scaling parameter $\lambda_{FM}$ might tend to limit the malleability of Ca^2+^-CaM in favor of the extended conformation (initial configuration of the simulations). Decreasing $\lambda_{FM}$ leads to an increase in γ_c:e_ and a decrease in $\bar{\text{Rg}}$. This is because decreasing the weight of the memory potential favors the hydrophobic interactions among residues of the two domains of Ca^2+^-CaM and reduces the free energy barrier between the two major conformations of Ca^2+^-CaM (extended and collapsed forms); consequently, the system becomes more flexible and can shift easily between the extended and collapsed conformations.

### Single Memory Optimization Captures the Dynamics of Ca^2+^-CaM

Although the global fragment memory weight of $\lambda_{FM}=0.1$ resulted in a system that can sample the major two conformations of the Ca^2+^-CaM and $\bar{\text{Rg}}$ is within the experimental range (Seaton et al., 1985; Heidorn and Trewhella, 1988; Kataoka et al., 1991), the ratio between the collapsed and the extended populations γ_c:e_ did not match the experimentally measured value (Anthis et al., 2011). Therefore, finer tuning of the local memory parameters W_n_^m^ is required for reproducing γ_c:e_ in the abovementioned NMR experiment (Anthis et al., 2011).

Since the crystal structure of Ca^2+^-CaM (PDB ID: 1CLL) demonstrates a value of Rg that is closest to the experimentally measured values and does not bind a target, we first examined whether variation in single memory weight of this structure ($W_{n}^{1\text{CLL}}$) could give rise to the conformational changes of the Ca^2+^-CaM. We performed a series of molecular simulations using US with $W_{n}^{1\text{CLL}}$ = 1, 2, … , 9 and maintaining $\lambda_{FM}=0.1$ and $W_{n}^{\text{others}}=1$ (Table 2 Model II). From the PMF profiles and the probability distributions shown in Figure 5, we show that the Ca^2+^-CaM samples both the extended and collapsed conformations in all the models we explored. In general, with the increase of $W_{n}^{1\text{CLL}}$, stability of the collapsed state decreases and the barrier between the extended and collapsed states increases, hence an increase in $\bar{\text{Rg}}$ and decrease in γ_c:e_. Specifically, without tuning any other local memory weights, $W_{n}^{1\text{CLL}}=8,\;9$, and $\bar{\text{Rg}}\;$and γ_c:e_ match with experimental values (Seaton et al., 1985; Heidorn and Trewhella, 1988; Kataoka et al., 1991; Anthis et al., 2011) quite well (Table 2).

### Central Linker of the Ca^2+^-CaM Determines the Conformational Change of Ca^2+^-CaM

Although the X-ray crystallography experiment shows that the central linker that connects the two domains of Ca^2+^-CaM has a helical structure, numerous studies including NMR have shown its high flexibility in solution. To explore the relationship between the flexible linker and the dynamics of Ca^2+^-CaM in more detail, we carried out a series of US molecular simulations with the memory weight of the central linker of the Ca^2+^-CaM, $W_{2}^{1\text{CLL}}=1,2,\ldots ,9$ while keeping $\lambda_{FM}=0.1$ and $W_{n}^{\text{others}}$ = 1, and $W_{n}^{1\text{CLL}}$(n = 1,3) = 5 (Table 2 Model III). As shown in the PMF profiles (Figure 6), $W_{2}^{1\text{CLL}}$ shows a similar effect on the thermodynamic properties of the Ca^2+^-CaM; with the increase of $W_{2}^{1\text{CLL}}$, stability of the collapsed state decreases and the barrier between the extended and collapsed states increases, hence an increase in $\bar{\text{Rg}}$ and decrease in γ_c:e_ (Table 2). This is likely because the two globular domains of CaM consist of stable secondary structures (mostly α-helices) and the involving fragments are fully represented and heavily weighted by $W_{n}^{\text{others}}$ (n = 1, 3) in other 59 memories, whereas the central linker connecting the two domains of CaM is modeled as an intrinsically disordered peptide; hence, the stability of the Ca^2+^-CaM is more sensitive to the parameter $W_{2}^{1\text{CLL}}$ corresponding to the central linker of CaM. Therefore, tuning $W_{n}^{1\text{CLL}}$ for the whole CaM is mostly equivalent to tuning $W_{2}^{1\text{CLL}}$ for the central linker. This can also be seen in the three highlighted models in Table 2, which reproduce the experimentally measure Rg and γ_c:e_ well (Seaton et al., 1985; Heidorn and Trewhella, 1988; Kataoka et al., 1991; Anthis et al., 2011).

In order to illustrate the conformational changes of Ca^2+^-CaM in solution, we selected representative snapshots from the simulations using the Ca^2+^-CaM model with $\lambda_{FM}=0.1$, $W_{n}^{\text{others}}$ = 1, $W_{1,3}^{1\text{CLL}}$ = 5, and $W_{2}^{1\text{CLL}}=8$, as highlighted in Table 2. The unstructured central linker of the extended structure (Figure 7D) unwraps and bends to allow the two domains of the Ca^2+^-CaM to interact with each other, as shown in Figure 7A–C). Figure 7A represents a completely collapsed structure similar to the compact structure (PDB ID: 1PRW) or in CaM-target complexes, however, the chance for Ca^2+^-CaM to sample this structure is low. Figure 7B represents a collapsed structure. Figure 7C represents a structure in the transition state between the collapsed and extended states.

### Coordination Geometry Is Necessary for Representing Divalent Ions in Coarse-Grained Models

Although it can be tempting to use the available intrinsically disordered protein (IDP) force fields such as AWSEM-IDP (Wu et al., 2018) (due to the disordered nature of the central linker) to study the dynamics of Ca^2+^-CaM, the heterogeneity of CaM needs to be considered. The high rigidity of the two domains upon calcium-binding limits the use of IDP force fields, thus making the computational investigation of CaM even more complex.

We further manifest the importance of coordination chemistry by comparing the three approaches to modeling Ca^2+^ as described in Method *Modeling Ca* ^*2+*^ *Ions in ASWEM Force Field* in Figure 8. With approach I, the Ca^2+^ charges were evenly distributed to all the negatively charged residues (Tsai et al., 2016) and Ca^2+^-CaM presents an equal probability of sampling the completely collapsed conformation and the extended conformation (γ_c:e_ = 1.01). $\bar{\text{Rg}}\;$∼ 18.64 Å is well below the experimental values (Seaton et al., 1985; Heidorn and Trewhella, 1988; Kataoka et al., 1991). With approach II by applying constraints on the Ca^2+^ loops, Ca^2+^-CaM samples favorably the completely collapsed conformation with Rg ∼ 15 Å (γ_c:e_ = 22.87). Consequently, the binding selectivity of Ca^2+^-CaM could be impeded due to the lack of the flexibility of calcium-binding loop in the system. Being confined in this completely collapsed conformation, Ca^2+^-CaM lacks the capacity of recognizing, binding, and activating the targets. With approach III (w/coordination chemistry), Ca^2+^-CaM samples both extended and collapsed conformations while preferring the former.

Force field development for Ca^2+^ ion is mostly limited to the aqueous solution rather than in protein-bound environments (Probst et al., 1985). One of the main challenges in computer simulations of macromolecules remains the development of force fields with the appropriate implementation of the metal ion (Gifford et al., 2007; Duarte et al., 2014; Li and Merz, 2017; Liao et al., 2017; Fracchia et al., 2018; Huang and MacKerell, 2018). Due to the high sensibility of the molecules upon bivalent metal ion binding, taking the Ca^2+^-induced conformational changes in CaM as an example, a lack of appropriate approach to model its effects on the molecules could impede the capability of the target molecule to transduce its signals, thus, leading to the inactivation and/or malfunction of the signaling pathways (Gaertner et al., 2004; Wang et al., 2011).

Most calcium-binding proteins such as CaM contain the EF-hand (helix-loop-helix) structural domain as the calcium-binding motif (Lepšík and Field, 2007; Capozzi et al., 2006). In this motif, the calcium ion is coordinated in a pentagonal bipyramidal configuration (Figure 3). The canonical positions 1, 3, 5, 7, 9, and 12 represent the positions of the residues involved in calcium coordination (Clapham, 2007; Gifford et al., 2007). Table 3 shows simple and efficient ways to consider the effect of the Ca^2+^ ions in the force field, where we distributed its charges to the negatively charged residues present in the pentagonal bipyramidal configuration. In contrast to other approaches where the harmonic constraint is applied to the calcium coordinated residues in the loop, or the charges are distributed to all the negatively charged residues in the system, our approach (III) not only considers the heterogeneity of CaM but also takes into account the coordination chemistry of calcium by giving the coordinated residues the freedom to participate to the different conformations of the system, which are relevant to the target selectivity of Ca^2+^-CaM.

### The Distribution of Ca^2+^-CaM Conformations Is Beyond the Extended and the Collapsed Structures

Ca^2+^-CaM undergoes substantial conformational changes upon binding to a target, in most cases, the Ca^2+^-CaM develops a collapsed form that wraps around the target peptide with the two domains coming in proximity (Meador et al., 1993; Westerlund and Delemotte, 2018; Tidow and Nissen, 2013). For various CaMBT molecules, CaM undergoes structural rearrangement to different degrees during the complex formation. Thus, the collapsed conformation of the intact Ca^2+^-CaM (e.g., Figure 7) assembles a vast variety of structures including a structure that resembles the compact conformation of a specific Ca^2+^-CaM/target complex (Figure 1D). This was also observed by X-ray crystallography (Fallon and Quiocho, 2003) and NMR studies (Anthis et al., 2011). This collapsed structure of the Ca^2+^-CaM (Figure 7A) confirms the earlier experiments that show multiple conformations of the Ca^2+^-CaM in solution, as well as the interaction between opposing domains of CaM (Johnson, 2006; Anthis et al., 2011; Her et al., 2018).

Previous studies have shown that the high flexibility of the linker in solution allows the two domains to independently undergo multiple conformations (Malmendal et al., 1999; Chou et al., 2001; Barton et al., 2002; Hoeflich and Ikura, 2002; Komeiji et al., 2002; Park et al., 2008; Wu et al., 2012). Structural differences can be inferred from crystallography, but the dynamical insights are pivotal to understand Ca^2+^-CaM interactions with the targets. Experimental and computational studies including NMR spectroscopy, X-ray crystallography, small-angle X-ray scattering, and molecular dynamics simulations unveil important details of the dynamics of Ca^2+^-CaM and paint a clear portrait of its conformational motions at the origin of the binding selectivity. The available structures of Ca^2+^-CaM show that it crystallizes into two major conformations: extended (Chattopadhyaya et al., 1992) and collapsed (Fallon and Quiocho, 2003), while in solution, Ca^2+^-CaM adopts multiple conformations (Project et al., 2006; Park et al., 2008; Wu et al., 2012; McCarthy et al., 2015; Fernandes and Oliveira-Brett, 2017; Jing et al., 2017; Her et al., 2018; Villalobo et al., 2018; Shimoyama, 2019). The conformational changes of CaM upon Ca^2+^ binding have been under intensive investigation (Weinstein and Mehler, 1994; Gilli et al., 1998; Project et al., 2006; Park et al., 2008; Wu et al., 2012). The goal of such studies is to understand the molecular mechanism that leads to the Ca^2+^-CaM target binding selectivity. How Ca^2+^-CaM interacts with its CaMBTs is of major interest. This presented community model of CaM allows transitions between a rich set of states beyond the two major extended or the collapsed conformations and are applicable for various Ca^2+^ bound conditions; this further could improve the interpretation of how target binding may tune CaM’s affinity for Ca^2+^ ions (Gaertner et al., 2004; Zhang et al., 2017).

## Conclusion

Here, we developed a “community model” of CaM that samples various conformations of CaM, incorporates various calcium-binding states, and carries the memory of binding with various targets. The model would be useful for studying the reciprocal relationship interplay between target binding and calcium binding through CaM’s conformational changes as the next step. We demonstrated a workflow of the development of the AWSEM model for CaM guided by physical knowledge and experimental data. Should new structures of CaM (or CaM/CaMBT complex) structures become available, the model can be easily expanded by including these structures in the memory library.

## Data Availability

The original contributions presented in the study are included in the article/Supplementary Material; further inquiries can be directed to the corresponding author.
