# Supplementary material for: Coarse-Grained Modeling and Molecular Dynamics Simulations of Ca2+-Calmodulin
Source: Front Mol Biosci. 2021 Aug 24;8:661322. doi: 10.3389/fmolb.2021.661322 (PMC8421859; doi:10.3389/fmolb.2021.661322)
Supplement: Supplementary file 1 [file Table1.DOCX]

Supplementary Material

# Supplementary Data

List of parameters used in the energy function

[Chain]

10.0 10.0 30.0

2.45798 2.50665 2.44973

[Shake]

10.0 3.83 2.36 2.71

[Chi]

20.0 -0.83

[Excluded]

5.0 3.0

5.0 4.0

[Excluded_P]-

4

1.0 3.0

1.0 4.0

[Excluded_R6]-

39.5935 4.0

39.5935 4.0

[Epsilon]

1.0

[Rama]

2.0

3

1.3149 15.398 0.15 1.74 0.65 -2.138

1.32016 49.0521 0.25 1.265 0.45 0.318

1.0264 49.0954 0.65 -1.041 0.25 -0.78

2.0 419.0 1.0 0.995 1.0 0.820

2.0 15.398 1.0 2.25 1.0 -2.16

[Rama_P]

3

0.0 0.0 1.0 0.0 1.0 0.0

2.17 105.52 1.0 1.153 0.15 -2.4

2.15 109.09 1.0 0.95 0.15 0.218

0.0 0.0 1.0 0.0 2.0 0.0

0.0 0.0 1.0 0.0 2.0 0.0

[SSWeight]

0 0 0 1 1 0

0 0 0 0 0 0

[ABC]

0.483 0.703 -0.186

0.444 0.235 0.321

0.841 0.893 -0.734

[Dssp_Hdrgn]

1.0

0.0 0.0

1.37 0.0 3.49 1.30 1.32 1.22 0.0

1.36 0.0 3.50 1.30 1.32 1.22 3.47 0.33 1.01

1.17 0.0 3.52 1.30 1.32 1.22 3.62 0.33 1.01

0.76 0.68

2.06 2.98

7.0

1.0 0.5

12.0

[P_AP]-

1.0

1.5

1.0 0.4 0.4

8.0

7.0

5 8

4

[Water]

1.0

5.0 7.0

2.6

10

2

4.5 6.5 1

6.5 9.5 1

[Burial]

1.0

4.0

0.0 3.0

3.0 6.0

6.0 9.0

[Helix]

1.2

2.0 -1.0

7.0 7.0

3.0

4

15.0

4.5 6.5

0.77 0.68 0.07 0.15 0.23 0.33 0.27 0.0 0.06 0.23 0.62 0.65 0.50 0.41

-3.0 0.35 0.11 0.45 0.17 0.14

0 -3.0

0.76 0.68

2.06 2.98

#[Fragment_Memory_Table]

scaling_factor

mem_file

gamma_file

rmin rmax dr

frag_table_well_width

fm_use_pre-computed_table_flag

fm_sigma_exp

[Fragment_Memory_Table]

0.1

multi_mem_2.mem

uniform.gamma

0 50 0.1

0.1

0

0.15

# The Debye-Hückel parameters

pair_style hybrid/overlay coul/debye 0.0127 3.5 vexcluded 2 3.5 3.5

pair_coeff * * vexcluded 0.0

pair_coeff 1 1 vexcluded 20.0 3.5 4.5

pair_coeff 1 4 vexcluded 20.0 3.5 4.5

pair_coeff 4 4 vexcluded 20.0 3.5 4.5

pair_coeff 3 3 vexcluded 20.0 3.5 3.5

pair_coeff 4 4 coul/debye 80

# Root mean square deviation (RMSD)

RMSD measures the degree of similarity from the trajectories with respect to the initial (or reference) structure by evaluating the square root of averaged sum of the squared differences of atomic distances. Its expression is given by the Equation S.1

where N is the total number of residues, $r_{ij}$ represents the instantaneous distance between atoms of residues *i* and *j*; $r_{ij}^{ref}$ is the same distance in the reference structure. We used the RMSD of the $C$ atoms to analyze the degree of similarity between our molecular dynamics structure and the initial structure (PDB ID 1CLL). Supplementary Figures 21 and 22 show the variation of the RMSD over the course of simulation from our final parameter’s optimization. The higher variability of the RMSD implies that the system visited the two major conformations, extended and collapsed.

# Supplementary Figures


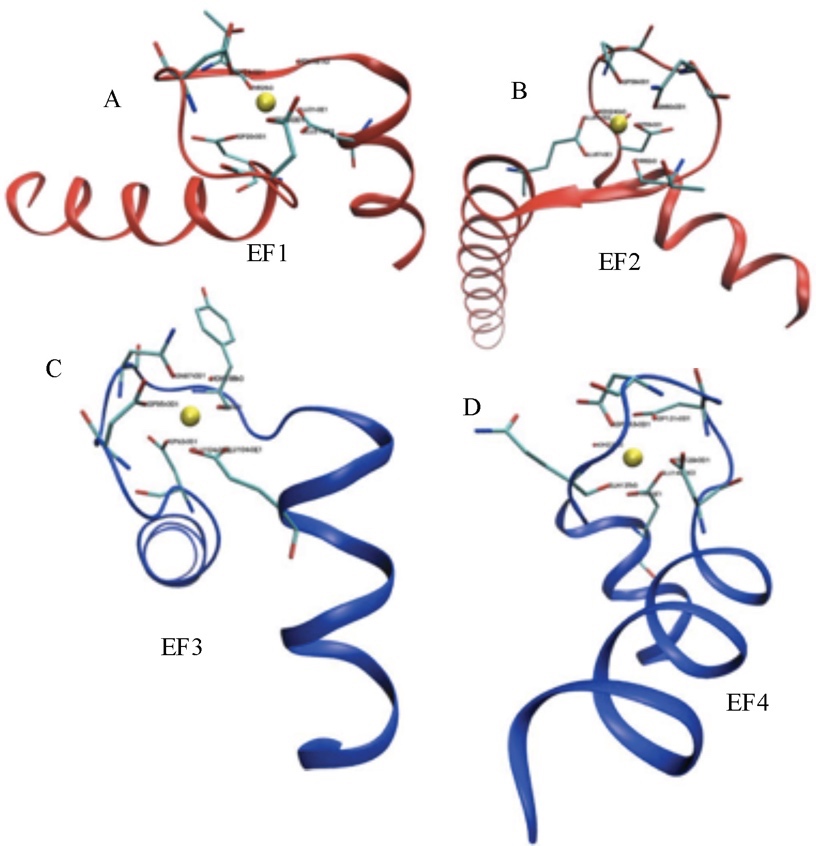


**Supplementary Figure 1.** EF hand structures of the Ca^2+^ binding loops in CaM. (**A**) EF-loop 1; (**B**) EF-loop 2; (**C**) EF-loop 3; (**D**) EF-loop 4.

**
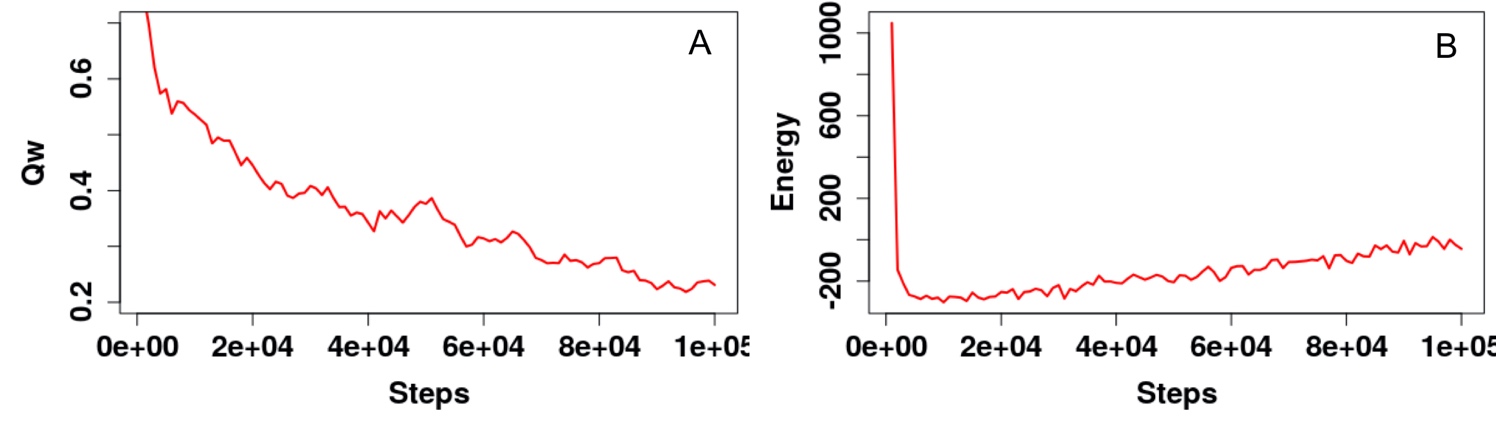
**

**Supplementary Figure 2.** Time variation of the order parameter $Q_{w}$ (**A**) and the associated energy (**B**) of the denatured CaM before the simulated annealing process. The unit of the energy is kcal/mol.


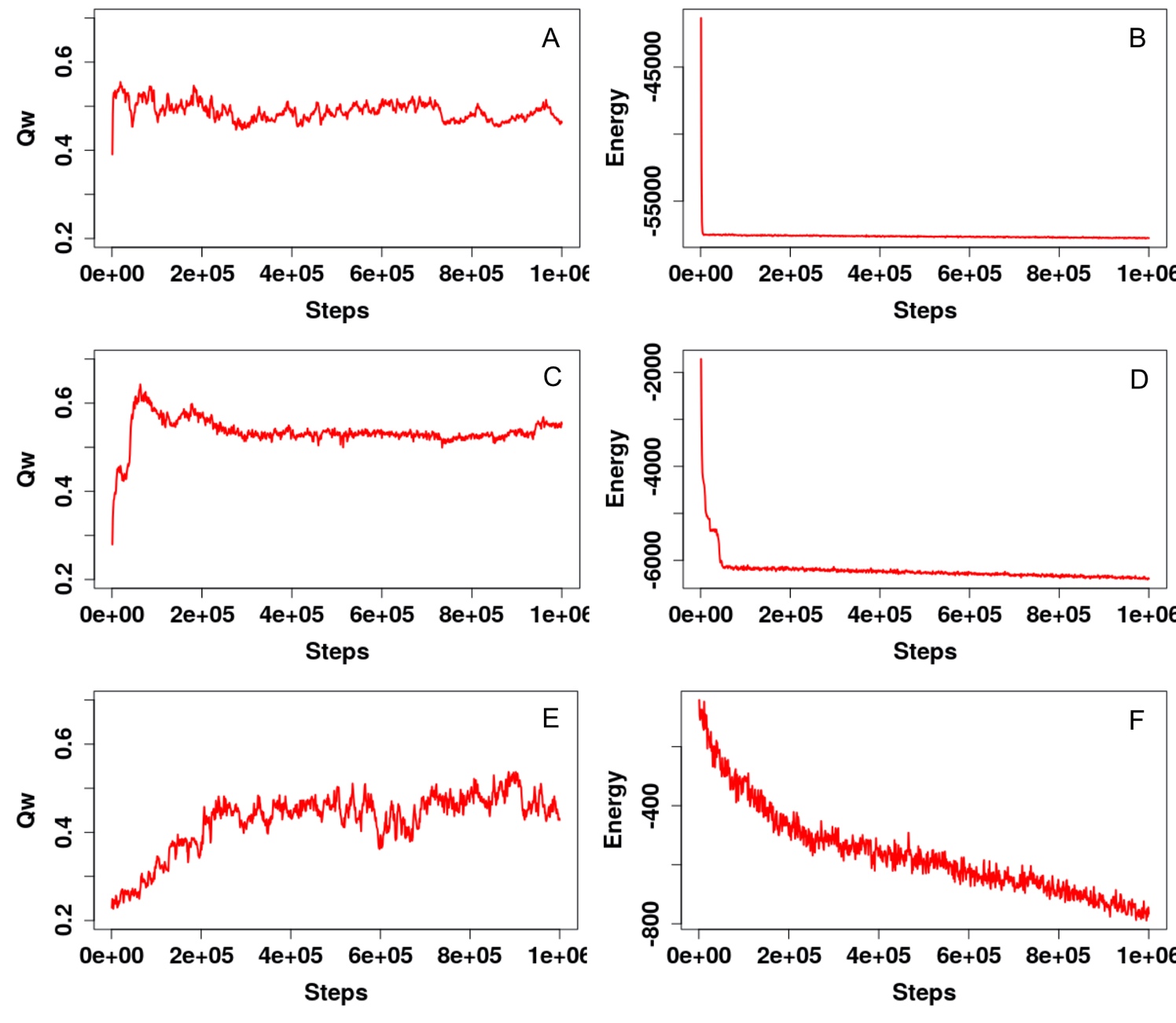


**Supplementary Figure 3.** Time variation of the order parameter $Q_{w}$ and the energy obtained from the simulated annealing of CaM using $\lambda_{FM}=1$ (**A**, **B**); $\lambda_{FM}=0.1$ (**C**, **D**); $\lambda_{FM}=0.01$ (**E**, **F**). The unit of the energy is kcal/mol.


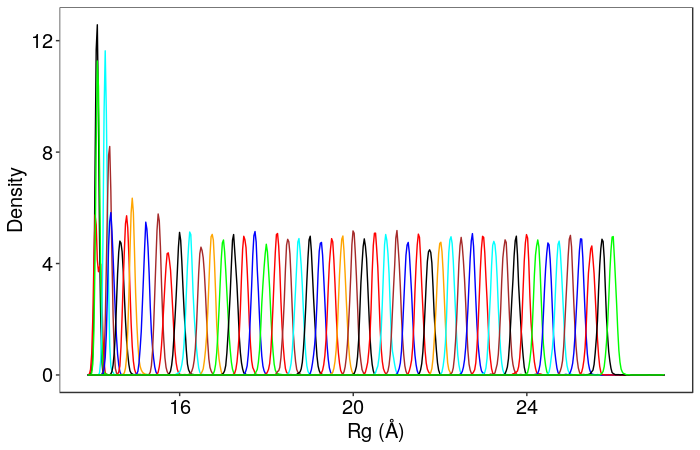


**Supplementary Figure 4.** Overlap in the distribution of Rg of the 53 consecutive windows. R_g_ was varied from 13 to 26 Å in increment of 0.25 Å. Each color represents the density plot of a single window. Simulations were carried out for 2,000,000-time steps using $\lambda_{FM}$ = 0.1, $W_{n}^{\mathrm{others}}$ =1, $W_{n}^{1CLL}$ = 5.


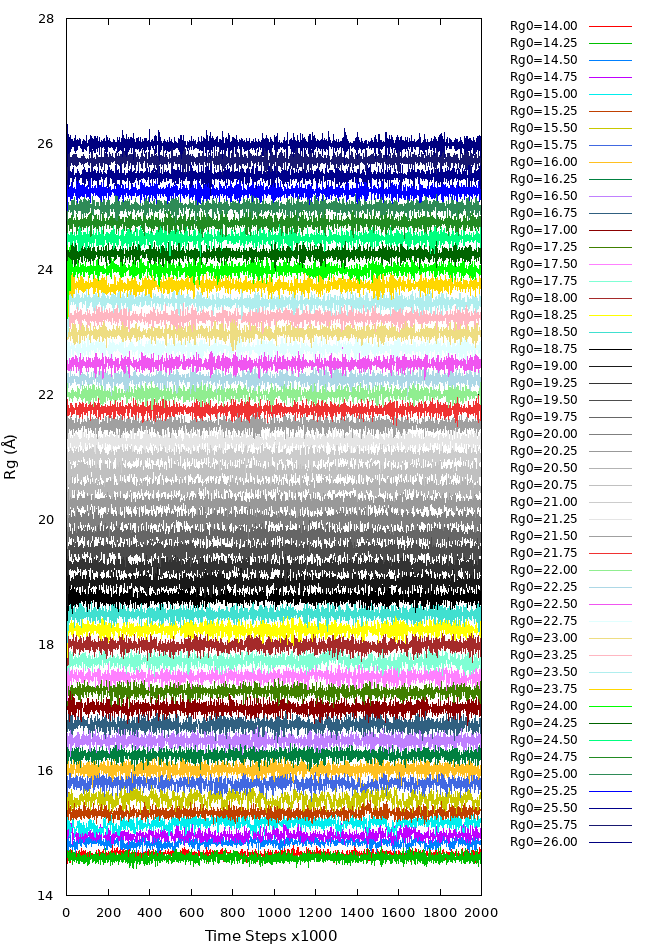


**Supplementary Figure 5.** Rg variation for all the windows in the Umbrella Sampling simulations. Each color represents the time variation of the Rg of a single window with the specified biasing Rg_0_. Simulations were carried out for 2,000,000-time steps using $\lambda_{FM}$ = 0.1, $W_{n}^{\mathrm{others}}$ =1, $W_{n}^{1CLL}$ = 5.


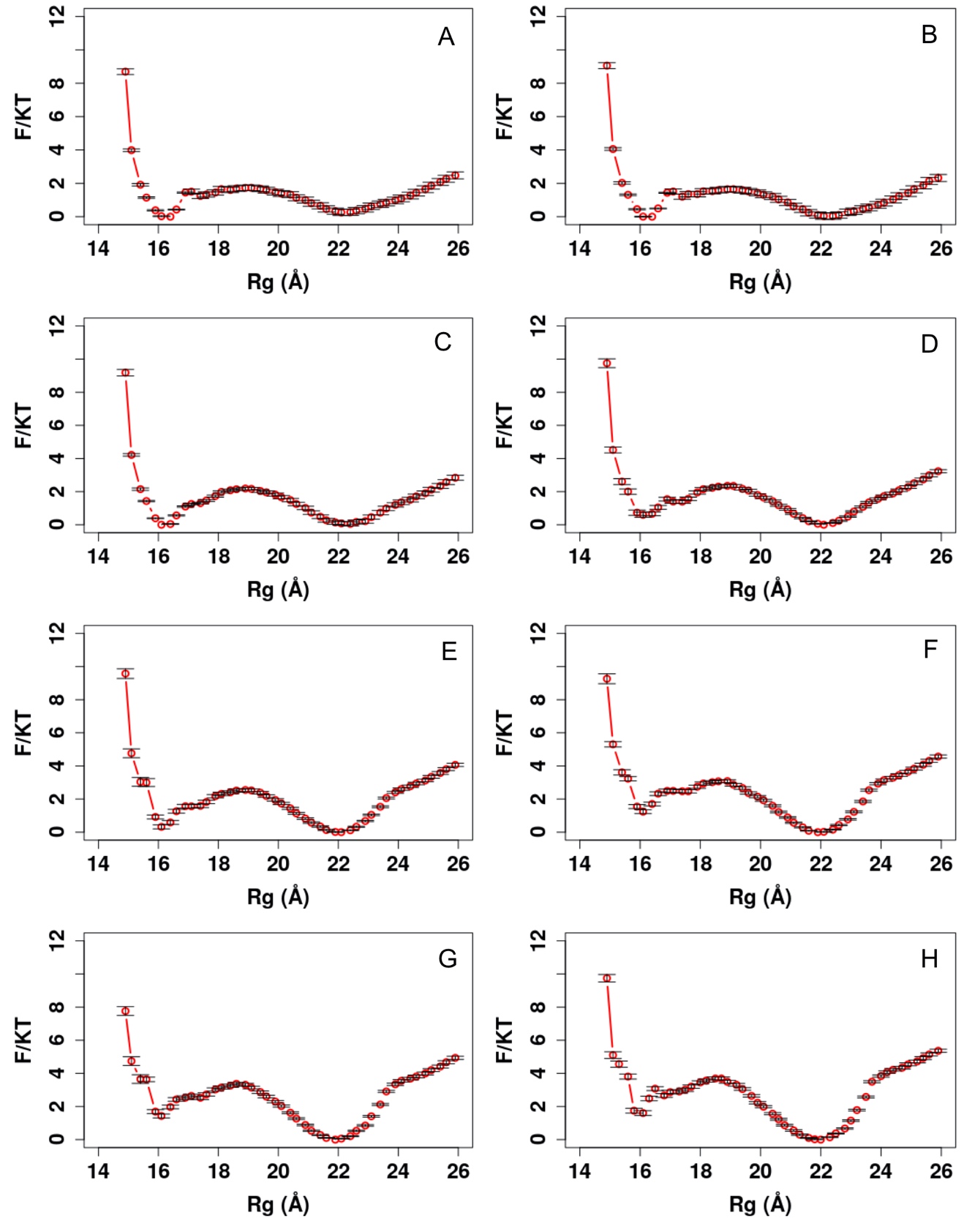


Supplementary Figure 6. PMF of the Ca^2+^-CaM with different memory parameters for memory 1CLL. The simulations temperature was T= 300K; $\boldsymbol{\lambda}_{\boldsymbol{FM}}$ = 0.1, $\boldsymbol{W}_{\boldsymbol{n}}^{\mathbf{others}}$ =1, (A – H) show the free energy profile along the Rg for each value of the $\boldsymbol{W}_{\boldsymbol{n}}^{\mathbf{1CLL}}$ = 2, 3, …9, respectively.


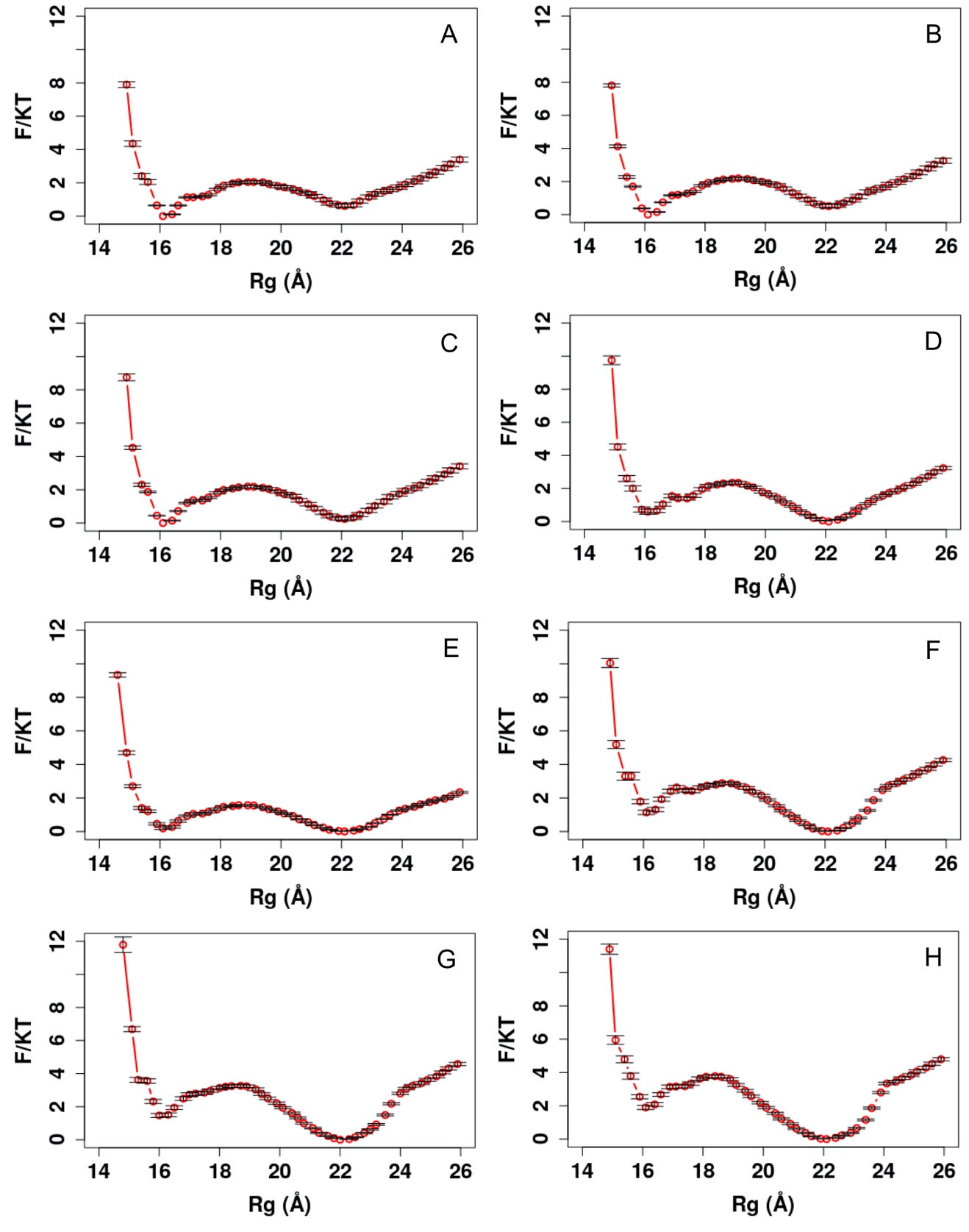


Supplementary Figure 7. PMF of the Ca^2+^-CaM with different memory parameters for the central linker of memory 1CLL. The umbrella sampling simulations were performed using the AWSEM protein force field with T= 300K, $\boldsymbol{\lambda}_{\boldsymbol{FM}}$ = 0.1, $\boldsymbol{W}_{\boldsymbol{n}}^{\mathbf{others}}$ =1, $\boldsymbol{W}_{\boldsymbol{1}}^{\mathbf{1CLL}}$ = 5 $\boldsymbol{W}_{\boldsymbol{3}}^{\mathbf{1CLL}}$= 5, $($A – H) show the free energy profile along the radius of gyration for each value of the $\boldsymbol{W}_{\boldsymbol{2}}^{\mathbf{1CLL}}$ = 2, 3, …9, respectively.
